# Social and Demographic Effects of Anthropogenic Mortality: A Test of the Compensatory Mortality Hypothesis in the Red Wolf

**DOI:** 10.1371/journal.pone.0020868

**Published:** 2011-06-23

**Authors:** Amanda M. Sparkman, Lisette P. Waits, Dennis L. Murray

**Affiliations:** 1 Department of Biology, Trent University, Peterborough, Ontario, Canada; 2 Department of Fish and Wildlife Resources, University of Idaho, Moscow, Idaho, United States of America; 3 Department of Biology, Trent University, Peterborough, Ontario, Canada; University of Pretoria, South Africa

## Abstract

Whether anthropogenic mortality is additive or compensatory to natural mortality in animal populations has long been a question of theoretical and practical importance. Theoretically, under density-dependent conditions populations compensate for anthropogenic mortality through decreases in natural mortality and/or increases in productivity, but recent studies of large carnivores suggest that anthropogenic mortality can be fully additive to natural mortality and thereby constrain annual survival and population growth rate. Nevertheless, mechanisms underlying either compensatory or additive effects continue to be poorly understood. Using long-term data on a reintroduced population of the red wolf, we tested for evidence of additive vs. compensatory effects of anthropogenic mortality on annual survival and population growth rates, and the preservation and reproductive success of breeding pairs. We found that anthropogenic mortality had a strong additive effect on annual survival and population growth rate at low population density, though there was evidence for compensation in population growth at high density. When involving the death of a breeder, anthropogenic mortality was also additive to natural rates of breeding pair dissolution, resulting in a net decrease in the annual preservation of existing breeding pairs. However, though the disbanding of a pack following death of a breeder resulted in fewer recruits per litter relative to stable packs, there was no relationship between natural rates of pair dissolution and population growth rate at either high or low density. Thus we propose that short-term additive effects of anthropogenic mortality on population growth in the red wolf population at low density were primarily a result of direct mortality of adults rather than indirect socially-mediated effects resulting in reduced recruitment. Finally, we also demonstrate that per capita recruitment and the proportion of adults that became reproductive declined steeply with increasing population density, suggesting that there is potential for density-dependent compensation of anthropogenically-mediated population regulation.

## Introduction

The effects of human-caused mortality on the population dynamics of wild species has long been a topic of both theoretical and management interest, and continues to be relevant for protection of small and recovering populations and establishment of sustainable targets for harvest. A major debate has centered on whether anthropogenic mortality is additive to natural mortality, resulting in a net reduction in total survival rates, or whether increased anthropogenic mortality is compensated for by a reduction in natural mortality, leaving the total survival rate unchanged [1]. In the latter scenario, compensation should be manifest through density-dependent mechanisms, where anthropogenic mortality triggers a release of limited resources that can enhance survival and recruitment in the remaining population, thereby buffering any negative effects on population growth. Empirical evidence suggests that compensation only occurs up to a certain threshold of anthropogenic mortality, above which a population can no longer sustain a stable or increasing growth rate, and begins to decline (e.g., [2]–[5]). However, the threshold at which compensatory mechanisms become insufficient and anthropogenic mortality becomes additive is a matter of intense debate (e.g., [6]–[9]), with recent studies in large carnivores suggesting that in some cases anthropogenic mortality can in fact be largely or fully additive [10]–[12].

Major mechanisms proposed for compensation in large carnivores, should it occur, include increased natural survival rates, increased birth rates and/or recruitment, and (in a metapopulation context) increased immigration or reduced emigration [7], [8], [13], [14]. However, evidence for the first two mechanisms is lacking, and it has been argued that where the effects of density are not strong and resources are not sufficiently limited, compensation via changes in natural survival and productivity rates will not necessarily occur [8], [12]. Furthermore, even at high densities, natural causes of death may not always be density-dependent (e.g., intraspecific strife, disease, or accident) [8]. In addition, the indirect, or sublethal effects of anthropogenic mortality remain largely unexplored, though there is evidence from several wolf populations that anthropogenic mortality can significantly disrupt natural social structure, and death of breeders can have important consequences for pup survival [15]–[18]. Thus, rather than benefiting from a general reduction in population density, productivity of breeding groups may suffer from loss of adult members, particularly in species with prolonged juvenile dependency.

In-depth studies of additive vs. compensatory mortality in wolves have primarily been conducted as meta-analyses incorporating data from diverse wolf populations worldwide with differing levels of population density and rates of immigration/emigration, and largely uncharacterized pedigrees and social dynamics (e.g., [7], [8], [12], [19]). While an inter-population meta-analysis can be a powerful tool for discerning broad-scale trends, analyses of single populations that can more thoroughly examine the roles of key factors such as population density and social dynamics on population-level responses to anthropogenic mortality are sorely needed. We explored the direct and indirect consequences of anthropogenic mortality in a reintroduced population of the red wolf (*Canis rufus*). Once distributed throughout the southeastern United States, the red wolf was declared extinct in the wild in 1980 but was successfully reintroduced into the Alligator River National Wildlife Refuge in North Carolina in 1987, as a result of a captive breeding program [20], [21]. Though a protected species, the red wolf is subjected to high levels of anthropogenic mortality from poaching, vehicle collision, and selective removal [22]. As this is the sole free-ranging population of the red wolf, it is an ideal system for examining the reproductive and survival effects of anthropogenic mortality unobscured by immigration of new wolves into the study area. Extensive monitoring of the reintroduced population since its inception has provided invaluable information on times and causes of death, population-wide pedigree, pack social dynamics, and estimates of population size. Using this information, we were able to explore the effects of anthropogenic mortality on natural and total survival rates, preservation of social bonds, reproductive success, and ultimately, population growth during periods of both high and low population density.

We hypothesized two main mechanisms through which anthropogenic mortality could affect population growth rate. First, anthropogenic mortality could have direct consequences for the number of adults in the population from one year to the next, either by directly reducing survival rates of existing adults or juveniles that would otherwise have been recruited into adulthood. Second, anthropogenic mortality could also exert indirect, sublethal effects on recruitment in so far as it results in loss of one or both members of a breeding pair, both of which normally provide care for pups [12], [15]. Since this population of red wolves was at small, increasing densities from 1990–1998, but reached relatively high and stable levels from 1999–2006 ([21], Fig. S1), we also hypothesized that additive effects on population growth, should they occur, would be strongest during the time period with low population density, when possibilities for density-dependent compensation are low.

To test for evidence of additive vs. compensatory effects of anthropogenic mortality, we used standard methods of regressing annual anthropogenic mortality rate on annual natural mortality, total survival and population growth rates. We took the analysis one step further by testing for additive vs. compensatory effects of anthropogenic mate loss on the annual natural dissolution of breeding pairs, total preservation of breeding pairs and population growth rates. We also tested for differences in fall (6-month-old) litter size between stable and packs and those that transitioned to new breeders or disbanded following the death of an existing breeder, to determine whether increases in breeder mortality could have indirect consequences for pup recruitment. Finally to assess the potential for compensatory responses to anthropogenic mortality in the long term, we tested for an effect of population density on per capita pup recruitment and the proportion of reproductive adults.

## Methods

### Data Collection

Between 1990–2006, free-ranging red wolves were captured primarily via foothold traps, equipped with very high frequency (VHF) radio-collars and subsequently monitored intensively to gather detailed information on mortality, reproduction, and pack affiliation [22]. Annual wolf population size estimates for the recovery area were taken from U.S Fish and Wildlife Service records ([21], Fig. S1). As an estimate of the number of individuals residing within a discrete management area, annual population size was also considered an estimate of annual population density for the purposes of this study. We restricted our analyses to adults, defined as individuals of one or more years of age, as detailed information on numbers and survival of pups from birth was not available due to the difficulty of monitoring pups at an early age [7].

Radio-collared wolves were monitored every 3–4 days from the ground or via fixed wing aircraft. Animals found dead were promptly retrieved and cause of death ascertained (e.g., see [11]). Death was attributed to either natural (intraspecific strife, disease, malnutrition), anthropogenic (illegal take, vehicle collision, handling, wildlife damage control operations, selective removal and return to captivity), or unknown causes. Selective removal of animals generally occurred at the request of landowners when wolf home ranges were established in proximity to livestock and depredation events had occurred. The effects of management-related deaths such as handling and selective removal are not generally included in individual-based survival analyses. However, we considered these as anthropogenic deaths for our population-level analyses, since anthropogenic removal of animals from the population likely has functionally equivalent effects on annual survival, pack dynamics, and population growth rates, irrespective of its specific cause. However, the same trends were upheld even when management-related deaths are censored (see Table S1).

There were 175 mortality events from 1990–2005, with 61% of all mortalities attributed to anthropogenic causes, 20% to natural causes, and 19% to unknown causes. Similarly high levels of anthropogenic mortality in the gray wolf are described in Murray et al. 2010 [11]. To be conservative, deaths due to unknown causes were considered to be “natural” for the purposes our analyses; however, inclusion of unknown deaths in the anthropogenic sample provided qualitatively similar results (see Table S2). During the study period 86 (33% of monitored individuals) wolves were censored. If all of these actually represented mortality events, they would comprise 33% of all mortalities, which is less than censoring rates in recent wolf survival studies [11], [23]. Notably, the proportion and influence of censored individuals is not often detailed in survival analyses [24], and we ascertained that considering censored animals as anthropogenic deaths did not have qualitative effects on our results (see Table S3).

Our detailed telemetry records allowed us to reconstruct social dynamics and behaviour of animals following mortality of other members of the social group. Instances of pair-bond dissolution were identified where one member of a breeding pair died, disappeared, or dispersed to a new location without his/her mate. There were 65 cases of pair bond dissolution during the study period, 42% due to anthropogenic deaths, and 58% due to natural causes (of these, 24% to natural death, 42% to dispersal, 11% to unknown death, and 24% to censoring).

Red wolves generally live in family groups with one breeding pair, older offspring, and pups [25]. Subsequent to the loss of one breeder, 48% of packs were disbanded and not replaced by new residents of their home range for one or more breeding seasons. The remaining 52% of packs retained the surviving breeder (who took on a new mate and recommenced reproduction after 0–3 breeding seasons), or rapidly transitioned to two new breeders. The number of recruits per litter in packs that were stable, with both parents remaining, or transitioned or ended following the loss of a parent within their first 6 months of life, was defined as the number of pups that survived from their spring birth to be counted by researchers the following fall (at approximately 6 months of age). The identity of breeding pairs and their corresponding offspring was determined from pedigree information for the population, as generated at 18 microsatellite loci via the program CERVUS 2.0 ([26]; for detailed genetic methods see [27]).

### Statistical Analyses

We used the Heisey-Fuller method to calculate estimates of annual survival and cause-specific mortality using telemetry data [28]; this method is equivalent to a piecewise exponential model and tends to perform well for wolf survival data derived from radio-telemetry, where intra-annual variability in hazards is negligible (e.g., [11], [23]). The annual rate of preservation of breeding pair bonds was calculated as the number of pair bonds preserved (i.e., not broken by anthropogenic or natural causes, such as natural death or dispersal), divided by the total number of pairs present in a given year. Annual rates of anthropogenic vs. natural dissolution of pair bonds were calculated as the number of pair bonds broken due to anthropogenic or natural causes, divided by the total number of pairs. The annual population growth rate, or finite rate of increase (**λ**) for year *n*, was calculated as the estimated population size in the year *n+1* divided by the population size in year *n.*

All analyses were conducted using JMP 8.0.2 (SAS Institute Inc.). We used regression analysis to test for evidence of additive or compensatory effects of anthropogenic mortality on total survival and pair bond preservation. The effects of anthropogenic mortality were defined as fully compensatory if the slope of the regression (±95% CI) equalled 0, and fully additive if the slope overlapped −1 [29]. The annual survival rate and pair-bond preservation rate were regressed against mortality rates and pair-bond dissolution rate, respectively, for both natural and anthropogenic mortality rates. In addition, annual population growth rate was regressed against natural mortality rate and pair-bond dissolution rates, and anthropogenic mortality rate and pair-bond dissolution rates. Annual population density was included as a covariate for analyses of population growth rate, as the red wolf population grew over the study period, and population growth was highest early in the study while the population was at low density ([21], Fig. S1). Population density was also included as a covariate for analysis of annual pair bond preservation, since rates of pair bond preservation appeared to decline with density (see Table 1), but was excluded from the analysis of annual survival rate (*P*>0.1). To further test for evidence of a compensatory adjustment in natural rates in response to anthropogenic rates, natural mortality and pair-bond dissolution rates were regressed against anthropogenic mortality and pair-bond dissolution rates, respectively, with the expectation that a negative relationship would imply a compensatory response, and no relationship would imply additivity.

**Table 1:** Regression analyses of annual rates of survival and preservation of pairs in relation to rates of natural and anthropogenic mortality and their effects on pair bonds.

Response	Effects	df	*F*	*P*	slope
*Adult survival*	natural mortality	1,14	9.18	0.0090	−1.77 (−3.03, −0.51)
	anthropogenic mortality	1,14	76.74	<0.0001	−0.95 (−1.18, −0.72)
*Preservation of pairs*	natural pair dissolution	1,13	8.77	0.0103	−0.91 (−1.57, −0.25)
	anthropogenic pair dissolution	1,13	33.39	<0.0001	−1.00 (−1.37, −0.63)
	population density	1,13	12.27	0.0039	∼

Relevant slopes and 95% confidence limits are given.

To test whether additivty/compensation was occurring in the same manner at both high and low density time periods, period could not simply be included as a main effect in our models, as it was confounded with population density, which explained continuous variation in some response variables (see above). However, whether population density was included or excluded from a model with time period as an effect, there was no significant interaction between period and anthropogenic rates. Thus, there was no statistical evidence for differences in additivity/compensation between the two time periods. However, since there was very low power for detecting an interaction due to limited sample size (n = 9 years for the low density time period, n = 7 years for the high density time period), we conducted our analyses by time period as well as using both periods combined, to determine whether results were consistent at high and low density periods.

Model comparisons using Akaike's Information Criterion adjusted for small samples (AICc) were initially performed to assess whether linear or polynomial models provided the better fit for these regressions [30]. However, as linear models provided the best fit for all regression models without exception (ΔAICc>2), we do not present these results here. Durbin-Watson tests were performed for all regression analyses to test for potential autocorrelation between sequential data, but no evidence of serial autocorrelation was detected (all *P*>0.1).

We used mixed-model analysis to test for an effect of pack status—stable (n = 115 litters), transitioned due to breeder loss (n = 16), or ended due to breeder loss (n = 17)—on the number of pups recruited per litter. Sample sizes for the different groups were unbalanced, but a Brown-Forsythe test suggested no evidence for unequal variance (*P*>0.1). Since the analysis included 148 litters in total, some of which originated from the same breeding pair, dam was included as a random effect. Age of dam and population density were initially included as covariates, but as both were non-significant (*P*>0.1), they were excluded from the final analysis.

To assess the relationship between density and reproduction at a population level, and thereby the potential for compensation in the long term, we tested for a relationship between the annual recruited pup to adult female ratio, and the total number of adult females in the population in a given year (which is directly proportional to population size). We also compared the proportion of adult females and males that were reproductive during three, rather than two time periods, for finer resolution since sample sizes were sufficient: 1988–1993, just subsequent to reintroduction when the population was small; 1994–1998, when the population was steadily growing; and 1999–2007, when the population was high and relatively stationary (see [25]).

### Ethics Statement

The field work on red wolves was conducted solely by the U.S. Fish and Wildlife Service and all work and procedures conformed to national standards for wildlife handling [31].

## Results And Discussion

### Anthropogenic Effects on Annual Rates

**Figure 1:**
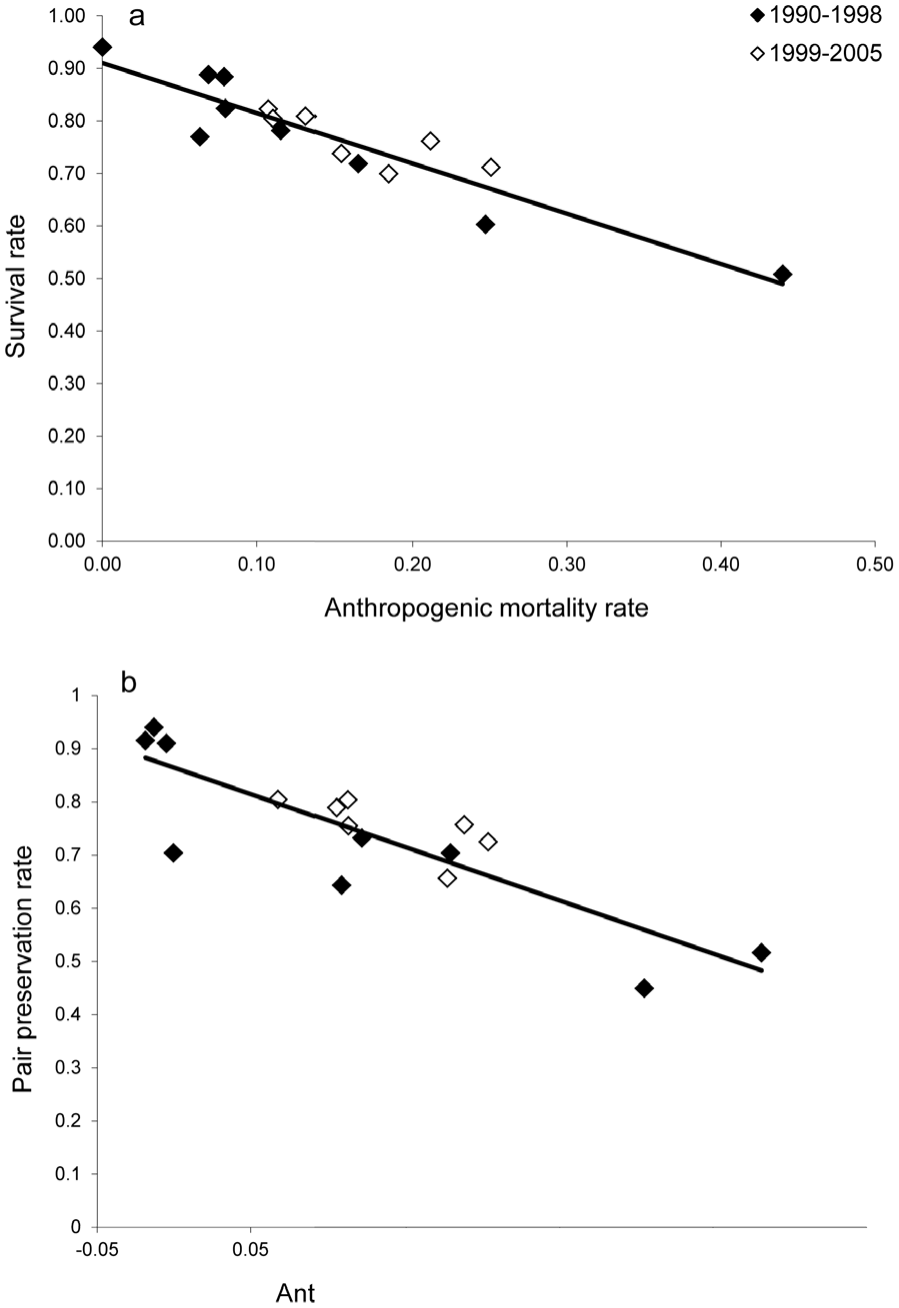
Annual survival rate (a) and pair preservation rate (b) for the red wolf. Values for (b) are corrected for density.

We found strong evidence that anthropogenic mortality is additive to natural mortality in the reintroduced red wolf population, with higher adult anthropogenic mortality resulting in lower annual adult survivorship (Table 1; Fig. 1A). These findings are consistent with recent studies in the gray wolf where anthropogenic mortality was found to be additive to natural mortality rates [11], [12]. Moreover, we also found evidence that anthropogenic mortality had an additive effect on the annual preservation of pair bonds, with higher levels of anthropogenic mate loss resulting in lower annual preservation of existing breeding pairs (Table 1; Fig. 1B). Lack of compensation in either survival or pair-bond preservation was further demonstrated by lack of a relationship between natural rates of mortality and pair-bond dissolution and their respective anthropogenic rates (Mortality—*F*_1,14_ *= *0.126; *P = *0.74; Pair-bond dissolution—*F*_1,14_ *= *0.085 ; *P = *0.78).

**Figure 2:**
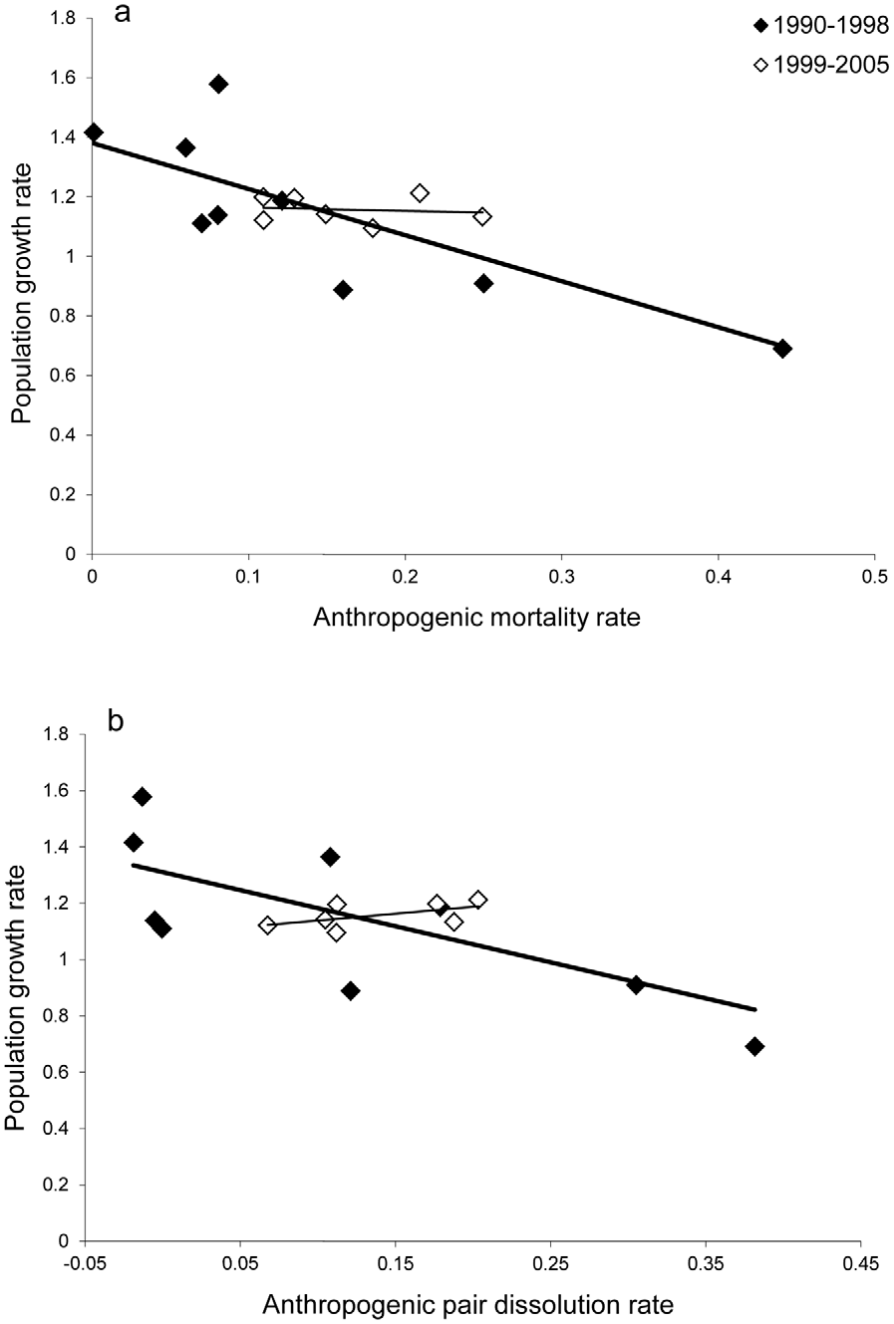
Annual population growth rate in relation to anthropogenic mortality rate of the red wolf. Model-corrected values are shown.

The additive effects of anthropogenic mortality itself translated to a linear decrease in population growth rate (Table 2; Fig. 2). Consistent with trends reported in meta-analyses of gray wolf populations [12], [18], this suggests that even low levels of anthropogenic mortality can play a constraining role in red wolf population growth. There was no evidence of a threshold at which anthropogenic rates transitioned from a compensatory to an additive effect, as has been suggested in other studies (e.g., [4], [5], [8]). However, one important caveat is that though there was no evidence of serial autocorrelation in our sample, additive trends for survival were largely driven by points representing the years 1990–1998, when the population was still actively growing (*F*_1,6_ *= *16.10; *P = *0.007; Fig 2), and were not evident between 1999–2005, when the population was relatively stationary (*F*_1,5_ *= *0.63 ; *P = *0.46; Fig. 2). Since anthropogenic mortality rates during 1999–2005 did not show as much variation as those during 1990–1998, this lack of a trend could be an artefact of small sample size. However, it is also possible that additive effects are stronger at lower density, when density-dependent effects are weaker, which would be predicted by theory.

**Table 2:** Regression analysis of annual population growth rate in relation to rates of natural and anthropogenic mortality and their effects on pair bonds.

Effects	df	*F*	*P*	slope
natural mortality	1,13	4.23	0.0605	−2.73 (−5.59, −0.13)
population density	1,13	23.97	0.0003	∼
natural pair dissolution	1,13	0.24	0.6336	∼
population density	1,13	22.57	0.0004	∼
anthropogenic mortality	1,13	18.38	0.0009	−1.55 (−2.33, −0.77)
population density	1,13	23.97	0.0003	∼
anthropogenic pair dissolution	1,13	11.99	0.0042	−1.28 (−2.08, −0.48)
population density	1,13	22.57	0.0004	∼

Relevant slopes and 95% confidence limits are given.

Nevertheless, strong additive anthropogenic effects on annual survival were evident during both time periods (1990–1998: *F*_1,7_ *= *43.11; *P<*0.001; 1990–2005: *F*_1,5_ *= *7.71; *P = *0.039), suggesting that if compensation at the level of population growth does occur at high density, it operates through mechanisms other than those considered here. The effects of anthropogenic mortality during periods of increasing and stationary trajectories within a single population have not been explored in detail in previous studies, so further research is clearly needed to test whether additivity is present to the same degree in both cases.

Remarkably, the regression of population growth rate on anthropogenic mortality rate (Fig. 1B) suggests a sustainable “harvest” (i.e., level of anthropogenic mortality above which **λ**<1, and the population begins to decline) of approximately 25%, which is comparable to estimates for the gray wolf, which have ranged from 20–30% [3], [7], [8], [12]. However, we should note that we have only one year in which the anthropogenic mortality rate exceeded this level, and additional points in the upper range could alter the slope of the regression. This lack of information on the effects of high levels of anthropogenic mortality also limits our present ability to determine whether, if there are indeed compensatory forces acting at high density (1999–2005), they become insufficient to sustain the population at anthropogenic mortality rates higher than 25%. Whatever the case, in populations such as this where population growth is the desired end of the reintroduction program, these findings suggest that anthropogenic mortality rates substantially lower than 25% are necessary to achieve positive growth rates, particularly at low population density.

### Mechanisms Underlying Additive Effects on Population Growth Rate

In an isolated population with no possibility of immigration from an adjoining population, there are two main mechanisms through which an additive effect of anthropogenic mortality could be manifest on population growth rate from one year to the next: (1) a direct reduction in numbers of existing adult and juveniles recruited to adulthood through mortality, and (2) an indirect reduction in recruitment of juveniles to adulthood through dissolution of social groups responsible for rearing pups. Since more adults die due to natural and anthropogenic causes combined than to natural causes alone, higher rates of direct anthropogenic mortality on adults will necessarily result in a decrease in population numbers, in so far as those numbers are not replenished through a compensatory response, such as reduced natural mortality or increased pup production and recruitment. As previously stated, there was no evidence of a compensatory response in adult natural mortality rates in the red wolf population. In the population at large, it may be that the high level of territoriality and discrete nature of packs make taking advantage of any localized death-related increase in resource availability in the short-term difficult for members of other wolf packs.

**Figure 3:**
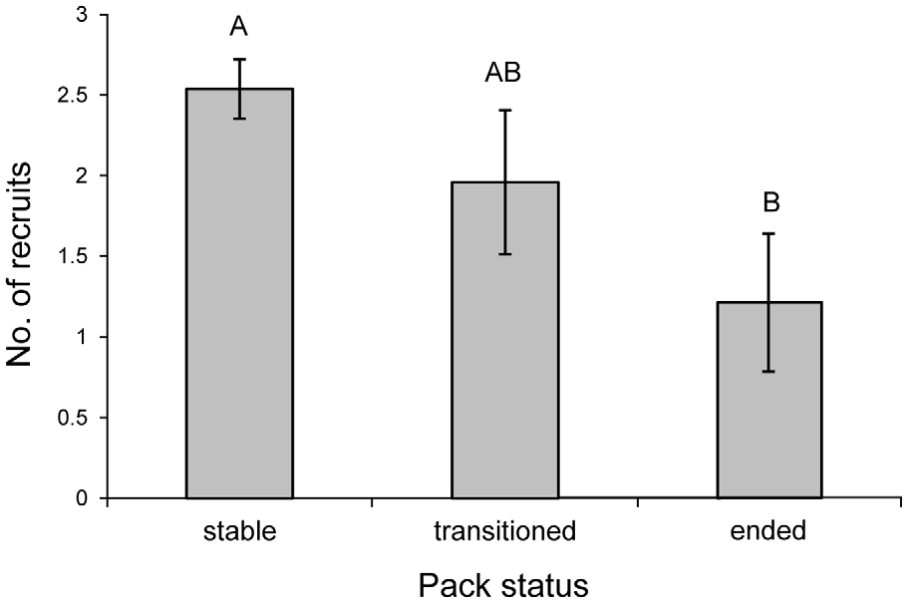
Least-square means and standard errors of the means of the number of red wolf recruits per litter according to pack status. Letters indicate significant differences from a post-hoc comparison of least-square means.

As for within-pack dynamics, we found that packs that are disrupted due to the death of a breeder showed decreased, rather than increased, numbers of pups recruited per litter (*F*_2,144_ *= *4.56; *P = *0.012; Fig. 3). Post-hoc analysis suggests that packs that were subsequently disbanded had the lowest number of recruits, and packs that retained the surviving breeder, or rapidly transitioned to a new breeding pair, had intermediate recruitment. Thus, within packs, any potential release of resources following the death of a breeder does not appear to compensate for the social benefits of having both parents present, and may slightly decrease in the absence of one parent. Furthermore, previous work has shown that the presence of older siblings in a red wolf pack increases pup survival at low density, and has no effect on pup survival at high density [25], suggesting that while competition for resources may occur among pack members, it may not always be as costly as is sometimes predicted. A positive association between pack size and pup survival following breeder loss has also been demonstrated in the gray wolf [15, but see 32]. Combined, this evidence suggests that per capita food availability is not a simple driver of survival and reproductive success, particularly in complex social systems.

Since anthropogenic mortality was responsible for 42% of breeding pair break-ups, and was additive to natural rates (Table 1; Fig. 1B), with almost half of all pair break-ups resulting in the disbanding of a pack, we hypothesized that reduced pup recruitment following anthropogenic loss of breeders could act as an indirect mechanism for additive effects of anthropogenic mortality on population growth rate. Indeed, the significant negative relationship between anthropogenic pair dissolution and population growth rate could be interpreted as support for this hypothesis (Table 2). Interestingly, however, the natural rate of pair dissolution, which includes dispersal as well as mortality events, was not significantly correlated to population growth rate (Table 2). This suggests that what might at first glance appear to be evidence for an additive effect of anthropogenic breeder loss on population growth may simply reflect a direct effect of loss of adults in general, and not indirect effects of breeder loss on recruitment per se. Thus, though here, as in other wolves, breeder loss by any means appears to have negative consequences for pack social structure and pup recruitment [15]–[17], and anthropogenic mortality can substantially add to these negative effects, the net effect of breeder loss on population growth rate appears to be negligible. One important point specifically relevant to this population, however, is that increases in breeder loss due to anthropogenic causes may also increase the probability of hybridization with coyotes (*Canis latrans*), which is currently a major threat to the recovery of the red wolf [21, Bohling and Waits, unpublished data].

### Long-Term Potential for Compensation

**Figure 4:**
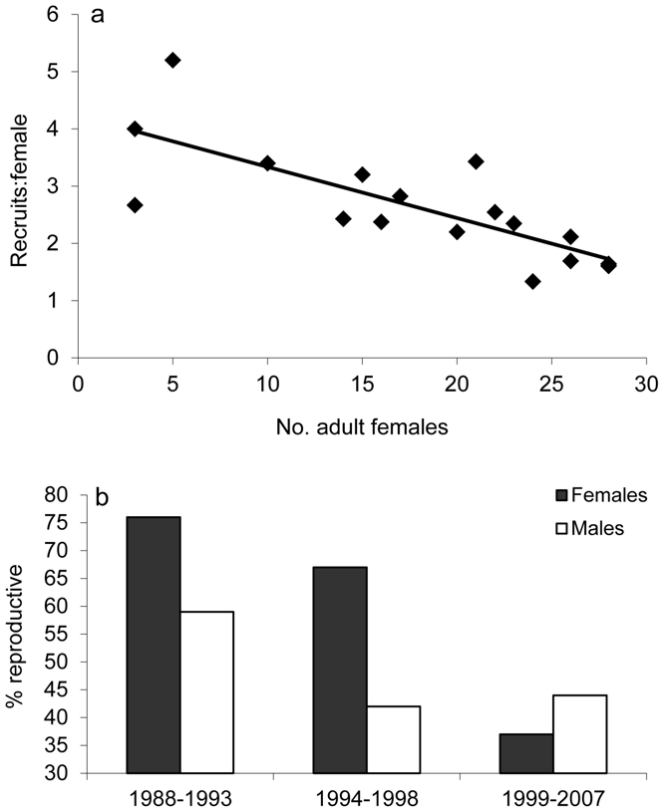
Effects of increasing population density over time on the reintroduced red wolf population. Annual ratio of recruits to adult females in relation to the total number of female red wolves over 2 years of age (a), and the proportion of male and female adults over 2 years of age that become reproductive in three different time periods (b).

Compensation is primarily predicted where there is evidence of negative effects of population density on survival and reproductive traits (e.g. [33]). In the red wolf population, we have previously demonstrated that key life-history traits such as lifetime reproductive success and survival from age 1 to 2 do show signs of being density-dependent [25], [34]. In this study we found a marked decline in the recruit-to-female ratio in relation to increasing numbers of adult females over time (*F*_1,17_ *= *14.44; *P = *0.001; Fig 4A). There are four potential explanations for this phenomenon: (1) increased anthropogenic mortality of pups with increasing density; (2) decreased pup productivity per female with density; (3) decreased pup recruitment per female with density; and/or (4) decreased proportion of reproductive females as density increases. We were unable to evaluate (1) – (2), as information on causes of death and survival rates of pups from birth was limited. As for (3), we found no evidence of an effect of density on recruited litter size. However, (4) alone is a highly plausible explanation, as there was a dramatic reduction in the proportion of adults that became reproductive as density has increased since reintroduction in 1987, declining from 68% to 41% of adults on average (n = 206; time period—χ^2^ = 9.02, *P = *0.0110; Sex— χ^2^ = 2.43, *P* = 0.119; Sex*time period— χ^2^ = 5.05, *P = *0.0800; Fig. 4B).

Given that only half of the packs with a single breeder lost to anthropogenic mortality managed to transition to a new breeding pair, and only 56% (n = 34) of these new pairs successfully produced a litter the following year, there is potential for a multi-year lag in productivity in a given home range following the dissolution of a breeding pair. However, the relatively low proportion of adults older than two years of age that become reproductive in this population at high density (Fig. 4A), and the decreasing per capita recruitment with increasing density (Fig. 4B) suggest that there is high potential for compensatory mechanisms to come into play in the long-term (i.e., within the next several years), if not in the short-term (i.e., within a single year). In other words, in spite of being additive, anthropogenic mortality of adults and its effects on breeding pairs and recruitment may not have substantial long-term ramifications due to an apparent superabundance of adults constrained from breeding due to density-related factors. The degree to which population density and/or anthropogenic mortality are responsible for the relatively stable size of the red wolf population from 1999–2006 is currently unknown. However, at this stage, the prevalence of non-breeding adults predicts high resiliency, given a release from either anthropogenic pressures and/or density-related constraints.

### Conclusion

We have provided evidence that anthropogenic mortality has additive effects on a reintroduced population of red wolves particularly at low density. These additive effects appear to be manifest through reduced annual survival translating to reduced population growth rate. We also found that packs disbanding after loss of a breeder show reduced numbers of recruits relative to stable packs. However, though anthropogenic mortality appears to have an additive effect on the annual rate of pair dissolution, there is little evidence that the disbanding of packs influences population growth rate per se, as natural rates of pair dissolution were not related to population growth. These findings are particularly interesting given that our population was essentially closed to dispersal, and thus our assessment of potential compensatory effects was restricted to on-site demographic changes. Finally, in spite of lack of evidence for compensation for direct anthropogenic mortality in the short term, the large proportion of non-reproductive adults at high density suggests potential for recovery from anthropogenic mortality in the long-term, as new breeding pairs take up residence in home ranges left vacant by disbanded packs.

## Supporting Information

Figure S1 **Annual red wolf counts in the Alligator River National Wildlife Refuge, North Carolina, from 1990–2006.** Data from U.S. Fish and Wildlife Service 2007.
(TIF)

Table S1 **Management-related deaths (due to handling or selective removal) censored.**
(DOC)

Table S2 **Unknown deaths considered as anthropogenic deaths.**
(DOC)

Table S3 **Censored individuals considered as anthropogenic deaths.**
(DOC)
