# Supplementary figures and images for: Social and Demographic Effects of Anthropogenic Mortality: A Test of the Compensatory Mortality Hypothesis in the Red Wolf
Source: PLoS One. 2011 Jun 23;6(6):e20868. doi: 10.1371/journal.pone.0020868 (PMC3121739; doi:10.1371/journal.pone.0020868)

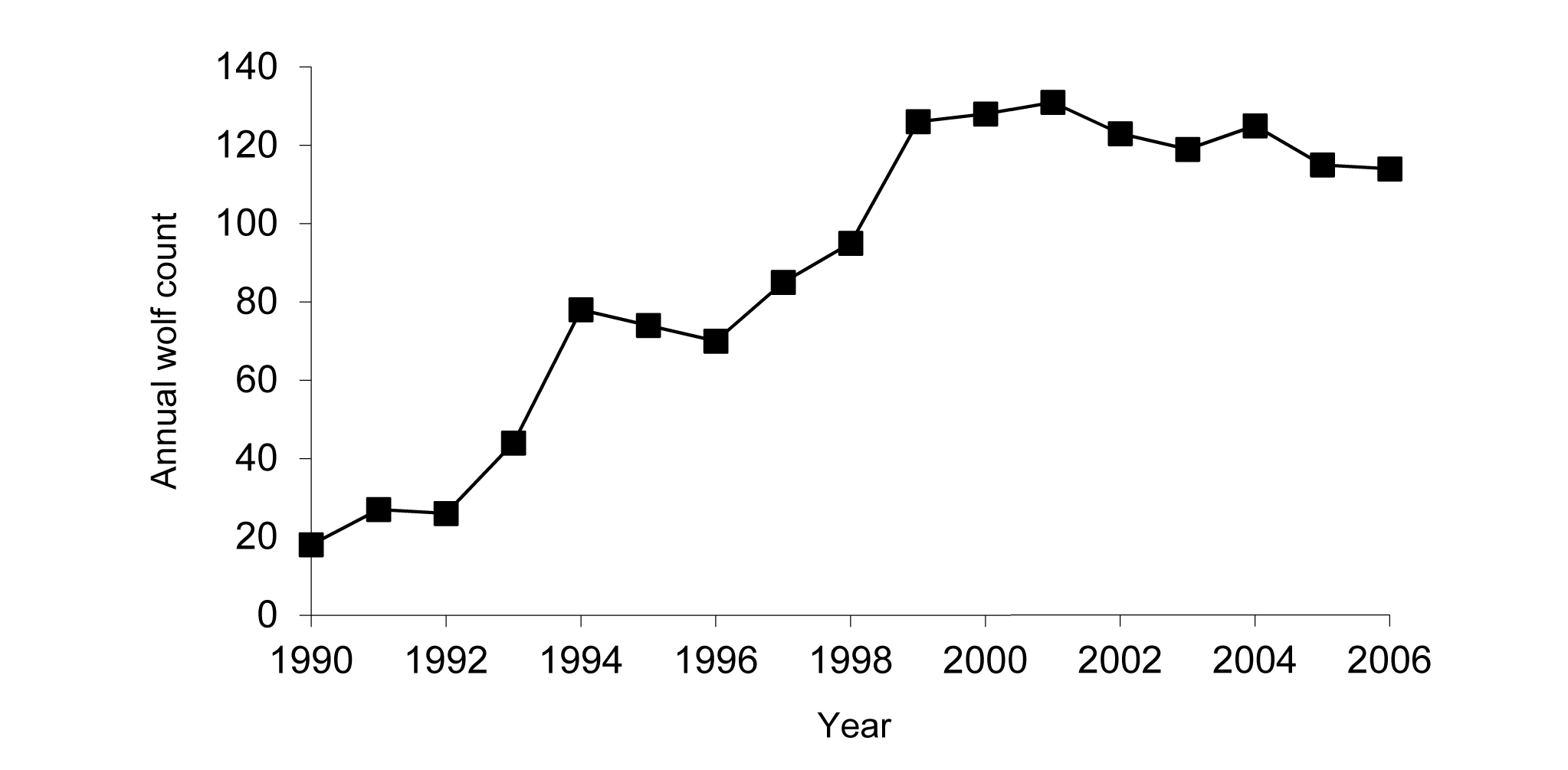

Supplement: Figure S1 — Annual red wolf counts in the Alligator River National Wildlife Refuge, North Carolina, from 1990–2006. Data from U.S. Fish and Wildlife Service 2007. (TIF) [file pone.0020868.s001.tif]
