# Supplementary material for: Social and Demographic Effects of Anthropogenic Mortality: A Test of the Compensatory Mortality Hypothesis in the Red Wolf
Source: PLoS One. 2011 Jun 23;6(6):e20868. doi: 10.1371/journal.pone.0020868 (PMC3121739; doi:10.1371/journal.pone.0020868)
Supplement: Table S1 — Management-related deaths (due to handling or selective removal) censored. (DOC) [file pone.0020868.s002.doc]

Table S1

| **Response** | **Effects** | **df** | ***F*** | ***P*** | **slope** |
| --- | --- | --- | --- | --- | --- |
| Annual survival rate | anthropogenic mortality | 1,14 | 19.99 | 0.0006 | -0.89 (-1.29,-0.49) |
| Population growth rate | anthropogenic mortality | 1,13 | 6.44 | 0.0260 | -2.12 (-3.90,-0.35) |
|  | population density | 1,13 | 5.16 | 0.0424 | ~ |
