# Supplementary material for: Social and Demographic Effects of Anthropogenic Mortality: A Test of the Compensatory Mortality Hypothesis in the Red Wolf
Source: PLoS One. 2011 Jun 23;6(6):e20868. doi: 10.1371/journal.pone.0020868 (PMC3121739; doi:10.1371/journal.pone.0020868)
Supplement: Table S2 — Unknown deaths considered as anthropogenic deaths. (DOC) [file pone.0020868.s003.doc]

Table S2

| **Response** | **Effects** | **df** | ***F*** | ***P*** | **slope** |
| --- | --- | --- | --- | --- | --- |
| Annual survival rate | anthropogenic mortality | 1,14 | 157.47 | <0.0001 | -1.05 (-1.23,-0.87) |
| Population growth rate | anthropogenic mortality | 1,13 | 19.44 | 0.0009 | -1.67 (-2.56,-0.86) |
|  | population density | 1,13 | 20.99 | 0.0006 | ~ |
