# Supplementary material for: Social and Demographic Effects of Anthropogenic Mortality: A Test of the Compensatory Mortality Hypothesis in the Red Wolf
Source: PLoS One. 2011 Jun 23;6(6):e20868. doi: 10.1371/journal.pone.0020868 (PMC3121739; doi:10.1371/journal.pone.0020868)
Supplement: Table S3 — Censored individuals considered as anthropogenic deaths. (DOC) [file pone.0020868.s004.doc]

Table S3

| **Response** | **Effects** | **df** | ***F*** | ***P*** | **slope** |
| --- | --- | --- | --- | --- | --- |
| Annual survival rate | anthropogenic mortality | 1,14 | 33.27 | <0.0001 | -0.80 (-1.11,-0.51) |
| Population growth rate | anthropogenic mortality | 1,13 | 13.39 | 0.0029 | -1.34 (-2.15,-0.55) |
|  | population density | 1,13 | 25.13 | 0.0002 | ~ |
